# “Wax On, Wax Off”: In Vivo Imaging of Plant Physiology and Disease with Fourier Transform Infrared Reflectance Microspectroscopy

**DOI:** 10.1002/advs.202101902

**Published:** 2021-08-02

**Authors:** Karina Khambatta, Ashley Hollings, Georgina Sauzier, Lilian M. V. P. Sanglard, Annaleise R. Klein, Mark J. Tobin, Jitraporn Vongsvivut, Mark R. Gibberd, Alan D. Payne, Fatima Naim, Mark J. Hackett

**Affiliations:** ^1^ School of Molecular and Life Sciences Curtin University Bentley Western Australia 6102 Australia; ^2^ Centre for Crop and Disease Management School of Molecular and Life Sciences Curtin University Bentley Western Australia 6102 Australia; ^3^ Infrared Microspectroscopy (IRM) Beamline ANSTO – Australian Synchrotron 800 Blackburn Road Clayton Victoria 3168 Australia

**Keywords:** epicuticular wax, infrared, microscopy, plant physiology, reflectance

## Abstract

Analysis of the epicuticular wax layer on the surface of plant leaves can provide a unique window into plant physiology and responses to environmental stimuli. Well‐established analytical methodologies can quantify epicuticular wax composition, yet few methods are capable of imaging wax distribution in situ or in vivo. Here, the first report of Fourier transform infrared (FTIR) reflectance spectroscopic imaging as a non‐destructive, in situ, method to investigate variation in epicuticular wax distribution at 25 µm spatial resolution is presented. The authors demonstrate in vivo imaging of alterations in epicuticular waxes during leaf development and in situ imaging during plant disease or exposure to environmental stressors. It is envisaged that this new analytical capability will enable in vivo studies of plants to provide insights into how the physiology of plants and crops respond to environmental stresses such as disease, soil contamination, drought, soil acidity, and climate change.

## Graphical abstract

Fourier transform infrared (FTIR) reflectance spectroscopic imaging has been used to image epicuticular waxes on the surface of plant leaves, in living plants, to observe how plant physiology changes in response to environmental stressors or disease.

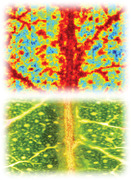

## 1 Introduction

Epicuticular waxes are located on the surface of many aerial plant parts and provide a vital range of protective functions to sustain plant health. Specifically, the wax coating serves to minimize water loss, protects against UV damage, provides a mechanical barrier to disease, and acts as an antifeedant, amongst others.^[^ ^1^, ^2^, ^3^ ^]^ Lipid synthesis and production of the epicuticular wax coating are therefore vital to plant health.^[^ ^1^, ^2^, ^3^ ^]^ Epicuticular waxes are chemically diverse, and numerous chemical species have been identified. In general, epicuticular waxes are a mix of long chain hydrocarbons of esters, alcohols, and ketones.^[^ ^2^, ^4^ ^]^ Wax synthesis is intrinsically linked to metabolism within the plant, hence unique differences in metabolism manifest in species‐specific epicuticular wax composition on the leaf surface.^[^ ^2^, ^4^, ^5^ ^]^ The composition of epicuticular waxes are influenced by seasonal variation, leaf age, and plant health.^[^ ^2^, ^3^, ^6^ ^]^ Therefore, these alterations in wax composition and distribution may serve as a marker of plant health and response to environmental stimuli.

High performance liquid chromatography (HPLC) and gas chromatography–mass spectrometry (GC–MS) techniques have been the standard analytical methods for characterizing the epicuticular wax composition of plant leaves. To obtain spatial information, using HPLC or GC–MS, requires incorporation of a spatial component into the sampling method, such as extraction of epicuticular waxes from the abaxial or adaxial leaf surface.^[^ ^2^, ^3^, ^4^ ^]^ HPLC and GC–MS approaches provide excellent chemical specificity and sensitivity (sub‐nM detection limits), however with some limitations. First, sample preparation is laborious and destructive and involves the use of organic solvent for wax extraction. Second, the spatially resolved information provided is limited to large, physically separable tissue structures rather than anatomical components such as leaf veins and stem.

To study leaf surfaces at the cellular or subcellular level, microscopic methods, are required. Techniques such as electron microscopy provide capability to image wax distribution on leaf surfaces, often at nanometer spatial resolution.^[^ ^7^, ^8^, ^9^ ^]^ However, the electron microscopy technique is destructive, incompatible with in vivo longitudinal studies, and provides only minimal information regarding chemical composition of the waxes.

More recently, vibrational microspectroscopy including Fourier transform infrared (FTIR) spectroscopy and Raman spectroscopy techniques have gained widespread use in plant research.^[^ ^7^, ^10^, ^11^, ^12^, ^13^, ^14^ ^]^ These methods do not offer the same level of spatial resolution as electron microscopy, nor the same chemical specificity and sensitivity as HPLC and GC–MS techniques. Nonetheless, FTIR and Raman microspectroscopy have been widely used as molecular characterization tools for mapping chemical functional groups at intermediate spatial resolution, typically 0.5–25 µm depending on instrument type, optics, and light source. In particular, FTIR and Raman techniques possess a distinct advantage allowing for direct and in situ measurement with minimal sample preparation, negating the need for solvent extraction of waxes from the leaf surface. Whilst FTIR and Raman microspectroscopy are often performed on thin sections, attenuated total reflectance (ATR)‐FTIR technique can be used to analyze the surface of the sample regardless of its thickness.^[^ ^15^ ^]^ The ATR‐FTIR method has become the most widely adopted mode of spectroscopic analysis of epicuticular waxes on leaf surfaces due to its simple sample preparation and the quick measurement approach.^[^ ^7^, ^10^, ^16^, ^17^ ^]^ However, the physical contact made between the ATR crystal and the leaf surface prevents the measurements to be repeated on the same location of the leaf, which therefore, renders ATR‐FTIR approach unsuitable for time‐course studies of living plants. More recent applications of vibrational spectroscopy to study epicuticular waxes include the use of atomic force microscopy infrared spectroscopy,^[^ ^13^, ^14^ ^]^ however although these techniques offered highly valuable chemical characterization at very high spatial resolution, physical or chemical extraction of the waxes from the plant was first required (i.e., the methods weren't compatible with in vivo imaging).

In this study, we have developed FTIR reflectance microspectroscopy to spatially resolve epicuticular waxes on the surfaces of plant leaves. The imaging protocol primarily takes advantage of specular reflection that occurs from the surface of thin films of ordered molecules at an interface. This has been previously established in the literature as infrared‐reflectance absorption spectroscopy (IRRAS) to obtain reflectance spectra from thin molecular films. The IRRAS effect is most pronounced when the film thickness is less than the wavelength of the light.^[^ ^18^, ^19^ ^]^ The percentage of reflected IR light in IRRAS is low (≈6%) resulting in a weak signal and low signal to noise (S/N) spectra, but although weak, the signal is sufficient for meaningful spectroscopic interpretations.^[^ ^18^, ^20^ ^]^ IRRAS spectra are typically presented as −log R/R0, where R is the reflectivity of the sample surface, and R0 is the reflectivity of the substrate.^[^ ^18^, ^20^, ^21^ ^]^ The reflectivity is depending on the angle of the incidence light source, the polarization of the light, and the molecular orientation at the surface, and this results in the possibility for both negative and positive “absorbance” bands to be observed in IRRAS spectra.^[^ ^18^, ^19^, ^20^, ^21^ ^]^ A range of substrates have been chosen, suitable for the background reflectivity measurements, ranging from reflective metal surfaces to H_2_O or D_2_O.^[^ ^18^, ^19^, ^20^, ^21^ ^]^ IRRAS is a well‐studied and characterized technique, previously used to study orientation of bio‐molecular films such as lipids and peptides at air–water interfaces.^[^ ^18^, ^20^ ^]^ Nevertheless, to our knowledge, the technique has not been demonstrated for in situ analysis of ordered molecules within biological tissues. This may be due to the physical nature of biological samples lacking thin, orientated molecular films capable of producing a sufficiently strong IRRAS effect.

Here, we report the use of FTIR microscopy and a linear array imaging detector configured in reflectance geometry to image epicuticular waxes at 25 µm spatial resolution across large areas of the leaf surface within a reasonable time frame (1–2 h). This capability enabled differentiation between major leaf anatomical structures (including stomata, veins, and stem), and has identified differences in epicuticular wax composition between young and mature leaves, and across seasons. Last, we demonstrate the capability of this method to monitor changes of epicuticular waxes on wheat leaves infected with a necrotrophic fungal pathogen, *Pyrenophora tritici‐repentis* (*Ptr*). The pathogen causes yellow spot disease, which has devastating impact on crop yields.^[^ ^22^, ^23^ ^]^ The plant–pathogen interactions induce detectable spectroscopic alterations to the wax layer. We also report for the first time non‐destructive longitudinal monitoring of epicuticular waxes on the leaf from a living plant using FTIR reflectance microspectroscopy. The study presented here demonstrates the unprecedented capability of the technique for studying plant epicuticular waxes in situ and in vivo, to gain a better understanding of plant physiological responses to environmental stressors.

## 2 Results

### 2.1 Non‐Destructive Imaging of Leaf Surfaces with Fourier Transform Infrared Reflectance Microspectroscopy

**Figure 1:**
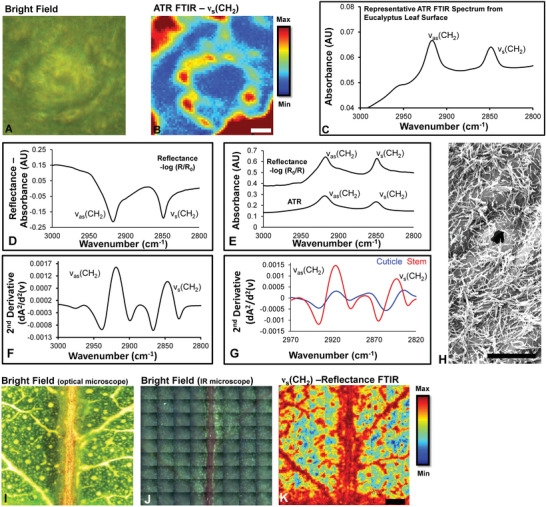
Epicuticular waxes on the leaf surface can be measured in situ by SR‐ATR‐FTIR spectroscopy (direct contact, destructive method) or by FTIR reflectance spectroscopy (non‐contact, non‐destructive). A) Bright field image of stomata on the surface of a *Eucalyptus* leaf, which was then analyzed by SR‐ATR‐FTIR spectroscopy. B) False‐color functional group images generated from integrated area under the curve for the *ν*_s_(CH_2_) absorbance band (2840–2865 cm^−1^) in SR‐ATR‐FTIR spectra showing location of wax rich regions. Scale bar = 5 µm. C) Representative SR‐ATR FTIR spectra of epicuticular wax. D) Representative FTIR reflectance spectra from the surface of the *Eucalyptus* leaf, and E) comparison of FTIR reflectance spectra with SR‐ATR FTIR spectra. F) Representative FTIR reflectance second derivative spectra. G) Different anatomical locations on the leaf surface produce different FTIR reflectance spectral signatures, which can be observed in the second‐derivative spectra. Spectra in (F) and (G) were not vector normalized prior to calculation of second‐derivatives. H) Representative SEM image captured from the surface of the leaf, showing thin wax structures that contribute to the infrared reflectance properties of the leaf surface. Scale bar = 10 µm. I,J) Bright field optical microscope (white light illumination) image of plant leaf surface, showing the sample region that was then imaged with FTIR reflectance (10× magnification). These bright field images of the leaf imaged with FTIR reflectance were collected on a dedicated optical microscope (I) and the optical microscope coupled to the FTIR spectrometer (J). K) False‐color FTIR reflectance functional group images of wax layer generated from second‐derivative intensity of the *ν*_s_(CH_2_) absorbance band at 2848 cm^−1^. Scale bar = 500 µm.

The process of sectioning enables imaging of interior leaf tissue structures that are not visible from the outer surface, such as lignin rich cell walls.^[^ ^7^, ^10^, ^24^, ^25^, ^26^, ^27^ ^]^ Sectioning unfortunately, can damage the exterior surface of the leaf sample and does not adequately preserve epicuticular wax distribution (Figure S1, Supporting Information). To prevent the damage to the leaf surface during sample preparation, the ATR‐FTIR technique is the most common approach among several other spectroscopic modes that allows FTIR spectra to be collected in situ from a sample surface. ATR‐FTIR mapping of *Eucalyptus* leaves revealed that wax distribution is heterogeneous relative to anatomical features such as stomata (**Figure** **1**A,B).^[^ ^16^ ^]^ The ATR‐FTIR spectra also revealed characteristic spectral features of waxes including intense *ν*_as_(CH_2_) and *ν*_s_(CH_2_) absorbance bands at 2930 and 2848 cm^−1^, respectively, without the presence of *ν*_as_(CH_3_) and *ν*_s_(CH_3_) modes (Figure 1C). Such features indicate that the waxes predominantly contain long chain aliphatic hydrocarbons. Despite the ability of ATR‐FTIR to directly map epicuticular waxes on leaf surfaces, the requirement for physical contact between the ATR crystal and leaf surface prevents longitudinal time‐course studies of the identical leaf region in living plants. FTIR reflectance microspectroscopy was therefore investigated as an alternative modality that may provide direct in situ analysis of epicuticular waxes compatible with longitudinal studies of plant development or disease progression.

Similar to the synchrotron ATR‐FTIR (SR‐ATR‐FTIR) spectra, FTIR reflectance spectra revealed the characteristic spectroscopic markers of waxes (i.e., intense *ν*_as_(CH_2_) and *ν*_s_(CH_2_) absorbance bands at 2930 and 2848 cm^−1^, respectively) with minimal evidence of *ν*_as_(CH_3_) and *ν*_s_(CH_3_) absorbance bands (Figure 1D,E). In particular, the *ν*_as_(CH_2_) and *ν*_s_(CH_2_) bands were found to display a strong negative signal in the FTIR reflectance spectrum (Figure 1D), or strong positive peaks in the second derivative spectra (Figure 1F), which is characteristic of IRRAS effect. This effect arises from increased specular reflectance for wavelengths of light at which an absorption band occurs, relative to wavelengths that do not correspond to a resonant absorbance. The intensity of the reflectance signal was found to vary across the leaf surface (Figure 1G) where wax crystals localize (revealed by scanning electron microscope [SEM], Figure 1H), providing a spectroscopic marker to image epicuticular wax in relation to leaf anatomy (Figure 1I–K). As can be seen in Figure 1I–K, FTIR reflectance imaging of the *ν*_s_(CH_2_) absorbance band revealed the location of key anatomical features of the leaf surface (stem, veins, and stroma tissue). The FTIR reflectance signal was drastically decreased in the leaf samples following incubation in ether, which extracted the surface epicuticular wax layer (Figure S2, Supporting Information).

### 2.2 Studying Seasonal Variations in Fourier Transform Infrared Reflectance Spectra of Leaf Surfaces

**Figure 2:**
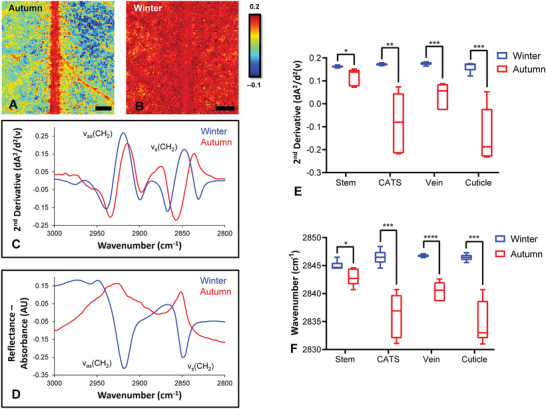
FTIR reflectance spectroscopic imaging reveals consistent spectroscopic differences associated with seasonal variation, comparing leaves analyzed in autumn against leaves analyzed in winter. A,B) False‐color functional group images of wax layer, generated from second‐derivative intensity of the *ν*_s_(CH_2_) absorbance band at 2848 cm^−1^, showing location of wax rich regions in leaves in autumn (A) and leaves in winter (B). Representative second‐derivative spectra (C) and non‐derivatized spectra (D) reveal the decreased reflectivity in spectra from leaves in autumn compared to winter, and show the shift of *ν*_s_(CH_2_) to lower wavenumbers in autumn compared to winter. E,F) Statistical analysis of replicates reveals that the observed differences in intensity (E) and peak positions (F) are significant. Data in (E) and (F) are presented as a box and whisker plots showing the mean, upper, and lower quartiles. Error bars show the standard deviation. All statistical testing was undertaken using a two‐way ANOVA, followed by post‐hoc testing, as described in the Experimental Section. Each experimental group consists of five replicates (*n* = 5). Post‐hoc testing was performed with *t*‐tests, corrected for four multiple comparisons using the Bonferroni method and an alpha of 0.05 (two‐tail testing). *p < 0.05, **p < 0.01, ***p < 0.001. Scale bar = 500 µm.

The FTIR reflectance signal showed reproducible seasonal variation. Images of leaves collected and analyzed during winter revealed a stronger reflectance signal of the *ν*_s_(CH_2_) mode relative to leaves collected in autumn (**Figure** **2**A–D). Analysis of the intensity of the *ν*_s_(CH_2_) reflectance signals (measured as second derivative intensity at 2848 cm^−1^), using five replicate leaves collected in autumn and winter, revealed a statistically significant effect of season on reflectance intensity (*p* < 0.05). Post‐hoc testing (Figure 2E) indicated that the reflectance intensity was significantly stronger in leaf tissue regions (stem, vein, cuticle, and cuticle adjacent to stem) in winter relative to those in the autumn (*p* = 0.03, *p* = 0.0006, *p* = 0.0005, and *p* = 0.002, respectively).

In addition to the increased reflectance intensity observed for leaves analyzed in winter relative to summer, an associated shift in the band positions was also observed. In particular, a shift to higher wavenumbers of *ν*(C—H) modes occurred in spectra collected from leaves in winter relative to those in autumn (Figure 2C). Two‐way ANOVA revealed that the effect was statistically significant (*p* < 0.0001). Post‐hoc testing (Figure 2F) indicated that the shifts in band position appeared to be significantly different between the samples in autumn and winter across the stem, vein, cuticle, and cuticle adjacent to stem tissue regions (*p* = 0.04, *p* = 0.00004, *p* = 0.0001, and *p* = 0.0005, respectively).

### 2.3 Variation in Fourier Transform Infrared Reflectance Spectra of Leaf Surfaces Display during Leaf Growth and Maturation

**Figure 3:**
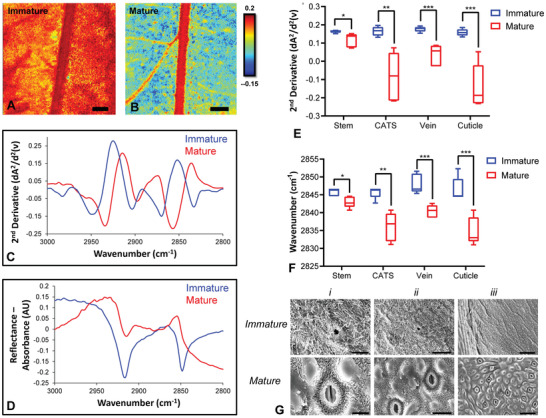
FTIR reflectance spectroscopic imaging reveals consistent spectroscopic differences between young (immature) and old (mature) plant leaves. A,B) False‐color functional group images of the wax layer, generated from second‐derivative intensity of the *ν*_s_(CH_2_) absorbance band at 2848 cm^−1^, showing location of wax rich regions in young (immature) leaves (A) and old (mature) leaves (B). Scale bar (A,B) = 500 µm. C) Representative second‐derivative and D) non‐derivatized spectra reveal the decreased reflectivity in spectra from older (mature) leaves, and show the shift of *ν*_s_(CH_2_) to lower wavenumbers in older (mature) leaves, relative to younger (immature) leaves. E,F) Statistical analysis of five replicates from different morphological regions show that differences in peak intensity (E) and peak position (F) are significant for stem, the cuticle adjacent to the stem (CATS), veins, and cuticle, when comparing spectra from young (immature) and old (mature) leaves. Data in (E) and (F) are presented as a box and whisker plots showing the mean, upper, and lower quartiles. Error bars show the standard deviation. All statistical testing was undertaken using a two‐way ANOVA, followed by post‐hoc testing, as described in the Experimental Section. Each experimental group consists of five replicates (*n* = 5). Post‐hoc testing was performed with *t*‐tests, corrected for four multiple comparisons using the Bonferroni method and an alpha of 0.05 (two‐tail testing). *p < 0.05, **p < 0.01, ***p < 0.001. G) Representative SEM images of surface or immature and mature leaves, showing that the immature leaf surface contains thin, fibril‐like wax structures, while large wax aggregates appear on the surface of mature leaves. Scale bar = 10 µm (*i*), 20 µm (*ii*), and 50 µm (*iii*).

To further investigate the sensitivity of FTIR reflectance microspectroscopy to changes in the epicuticular wax layer of leaves, the reflectance signals obtained from the surface of immature and mature *Eucalyptus* leaves were compared (**Figure** **3**A,B). Similar to the results observed with seasonal variation, changes in the intensity of the reflectance signal and position of the *ν*(C—H) bands was observed during leaf growth and maturation. Specifically, increased reflectance intensity and shifts to higher wavenumbers were observed for *ν*(CH_2_) absorbance bands on the surface of immature leaves relative to mature leaves (Figure 3C,D). Statistical testing confirmed that leaf growth had a significant effect on reflectance intensity (*p* < 0.0001) and peak position (*p* < 0.0001). The intensity of the *ν*_s_(CH_2_) band (measured at 2848 cm^−1^) increased in immature leaves across the stem, veins, cuticle, and cuticle adjacent to stem relative to mature leaves (*p* = 0.02, *p* = 0.0007, *p* = 0.0006, and *p* = 0.003, respectively) (Figure 3E). Likewise, the position of the *ν*_s_(CH_2_) band shifted to higher wavenumbers in the immature leaves relative to mature leaves (Figure 3F), which was observed in the spectra collected across the stem, veins, cuticle, and cuticle adjacent to stem (*p* = 0.01, *p* = 0.001, *p* = 0.0007, and *p* = 0.0009, respectively). SEM images revealed substantial differences in wax structure between immature and mature leaves, with thin fibril‐like structures observed in immature leaves, and larger agglomerates observed in mature leaves (Figure 3G).

**Figure 4:**
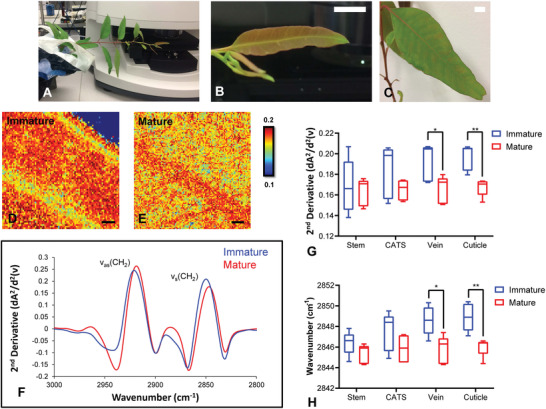
FTIR reflectance spectroscopy enables longitudinal monitoring of epicuticular waxes. A–C) Representative optical images of the leaf of *Eucalyptus* sapling imaged at different time points depicting young (B) and 7 weeks older (C). D,E) False‐color functional group images of wax layer, generated from second‐derivative intensity of the *ν*_s_(CH_2_) absorbance band at 2848 cm^−1^, showing increased intensity at 2848 cm^−1^ in the young leaf (D) compared to the same leaf 7 weeks later (E). F) Representative second‐derivative FTIR reflectance spectra showing the shift of the *ν*_s_(CH_2_) band to lower wavenumbers, associated with leaf maturation. G,H) Statistical analysis of five replicates reveals differences in intensity (G) and peak positions (H) are significant for vein and cuticle and not for stem and cuticle adjacent to the stem (CATS). Data in (G) and (H) are presented as a box and whisker plots showing the mean, upper, and lower quartiles. Error bars show the standard deviation. All statistical testing was undertaken using a two‐way ANOVA, followed by post‐hoc testing, as described in the Experimental Section. Each experimental group consists of five replicates (*n* = 5). Post‐hoc testing was performed with *t*‐tests, corrected for four multiple comparisons using the Bonferroni method and an alpha of 0.05 (two‐tail testing). *p < 0.05, **p < 0.01. Scale bar (B,C) = 1 cm; (D,E) = 500 µm.

To demonstrate that FTIR reflectance microspectroscopy holds the capability to monitor changes to plant epicuticular waxes in vivo, and is therefore compatible with longitudinal studies, we sought to reproduce the results described above in vivo. To do this, five leaves were imaged in the exact same region 7 weeks apart (**Figure** **4**). The results from in vivo FTIR reflectance imaging (Figure 4A–E) were found to be consistent with the ex vivo measurements. Importantly, the decrease of reflectance signals and shifts of the characteristic *ν*_s_(CH_2_) band to lower wavenumbers was reproduced during maturation of leaves as measured in vivo (Figure 4F). The results were confirmed to be statistically significant using two‐way ANOVA (*p* = 0.0006 and *p* < 0.0001 for signal intensity and band position, respectively). Post‐hoc testing revealed that the intensity of the *ν*_s_(CH_2_) band (measured at 2848 cm^–1^) increased in immature leaves across the veins and cuticle, relative to mature leaves (*p* = 0.02 and *p* = 0.002, respectively) (Figure 4G). Likewise, the position of the *ν*_s_(CH_2_) band shifted to higher wavenumbers in the immature leaves relative to mature leaves, in spectra collected across the vein and cuticle (*p* = 0.01 and *p* = 0.004, respectively) (Figure 4H). Differences between immature and mature leaves were not observed for the leaf stem or cuticle adjacent to stem.

### 2.4 Fourier Transform Infrared Reflectance Spectra of Diseased Wheat Leaves during Progression of Fungal Infection

**Figure 5:**
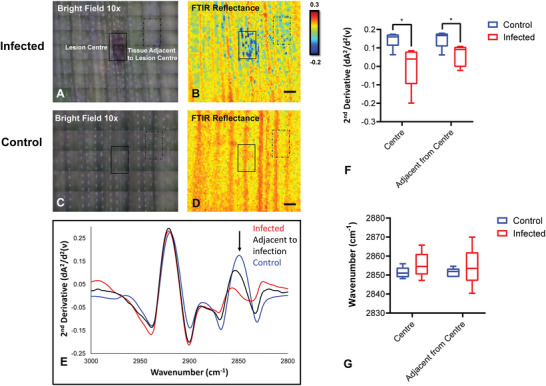
FTIR reflectance spectroscopy enables monitoring of changes in epicuticular wax induced during plant–pathogen interactions. A,C) Bright field optical microscopy images (10× magnification) of control (A) and yellow spot infected (C) wheat leaf samples. B,D) FTIR reflectance false‐color functional group images of wax layer generated from second‐derivative intensity of the *ν*_s_(CH_2_) absorbance band at 2848 cm^–1^ indicating differences between control (B) and infected (D) wheat leaf sections. Scale bar = 500 µm. E) Representative second‐derivative FTIR reflectance spectra show the shift of the *ν*_s_(CH_2_) band to higher wavenumbers closer to infected site. F,G) Statistical analysis of replicates reveals differences in intensity (F) but not in peak positions (G), between control and infected leaves. Data in (F) and (G) are presented as a box and whisker plots showing the mean, upper, and lower quartiles. Error bars show the standard deviation. All statistical testing was undertaken using a two‐way ANOVA, followed by post‐hoc testing, as described in the Experimental Section. Each experimental group consists of five replicates (*n* = 5). Post‐hoc testing was performed with *t*‐tests, corrected for four multiple comparisons using the Bonferroni method and an alpha of 0.05 (two‐tail testing). *p < 0.05.

To further extend our work in *Eucalyptus* leaves and to demonstrate the potential of the FTIR reflectance imaging method for studying waxes in an agricultural context, a set of infected (*n* = 5) and control (*n* = 5) wheat leaves were imaged (**Figure** **5**). Reflectance imaging was carried out in wheat leaves infected with *Ptr* as a case study, which showed consistent spectroscopic changes associated with varied wax distribution on the leaf surface. The SR‐ATR‐FTIR mapping technique was used to validate that fungal infection altered wax distribution on the surface of wheat leaves (Figure S3, Supporting Information). In the FTIR reflectance imaging data, a reduction of reflectance intensity and a subtle shift in position of the *ν*_s_(CH_2_) bands to higher wavenumbers were observed with increased proximity to the necrotic core (fungal infection resulting in dead tissue) (Figure 5E). Statistical testing confirmed that the reduction in reflectance intensity was significant (*p* < 0.01); however, shifts in the peak position were not significant. The reduction in intensity of the *ν*_s_(CH_2_) band (measured at 2848 cm^−1^) was found to be significant both at the necrotic core, and in tissue adjacent to the necrotic lesion (*p* = 0.03 and *p* = 0.03) (Figure 5E). The later finding is significant as they confirm the presence of an impacted asymptomatic region where no visual signs of infection were present (Figure 5A–D), and thus FTIR reflectance imaging technique was shown to be capable of detecting subtle chemical differences in epicuticular waxes before visible leaf damage occurred.

## 3 Discussion

In this study, we demonstrate for the first time that a sufficiently strong infrared reflectance signal is produced from the surface of plant leaves across the *ν*(C—H) region (3000–2800 cm^−1^, corresponding to light wavelengths of ≈3.3–3.6 µm). The detection of this signal enables characterization of epicuticular waxes. The spectroscopic features show intense *ν*_as_(CH_2_) and *ν*_s_(CH_2_) spectral features, but minimal contribution from *ν*_as_(CH_3_) and *ν*_s_(CH_3_) absorbance bands, which support long chain aliphatic wax as the main source of the reflectance signal. The strong negative peaks in the spectra for *ν*_as_(CH_2_) and *ν*_s_(CH_2_) vibrational modes support the proposition that the signal is originating from a thin‐film effect, known as one of IRRAS characteristic features. To strong negative signal results from increased reflectivity of light at these wavelengths, which indicates the presence of a thin molecular film, thinner than the wavelength of incident light,^[^ ^18^, ^19^ ^]^ which is possible given that the epicuticular wax layer is typically 50–200 nm thick.^[^ ^28^ ^]^ Therefore, owing to the strong IRRAS signal produced by the thin epicuticular wax layer on leaves, we demonstrate that FTIR reflectance microspectroscopy is sensitive to, and capable of imaging the epicuticular wax coating on leaf surfaces.

It is well‐established that the epicuticular wax coatings change as a consequence of seasonal variation and leaf maturation.^[^ ^2^, ^3^, ^6^ ^]^ In this study, we have demonstrated that the resulting FTIR reflectance signal from the leaf surface significantly changes in response to these events. It is rationalized that the epicuticular wax layer becomes thinner in winter, as there is less risk of plant dehydration, and that the decreased wax thickness enhances reflectance intensity, thus increasing the intensity of the negative peaks observed in the spectra. Likewise, young leaves that have reduced surface area and therefore reduced risk of water evaporation have been shown to have a thinner wax layer compared to mature leaves,^[^ ^28^ ^]^ which was further supported by SEM imaging in this study. This can account for the stronger FTIR reflectance signals observed in younger leaves relative to more mature leaves in this study. Critically, we were able to reproduce these observations when measuring leaf maturation in vivo, in a longitudinal study of leaf development. To our knowledge, this is the first report to demonstrate in vivo time‐course monitoring of changes to plant epicuticular waxes.

We have also observed shifts in the position of the *ν*(C—H) bands as a function of seasonal change or leaf maturation; however, further studies are required to determine the cause of these shifts, and fully determine what chemical information can be inferred from the changes (e.g., the shift of the *ν*_s_(CH_2_) to lower wavenumbers during leaf maturation). In traditional FTIR absorbance spectroscopy, it is widely accepted that a shift of the *ν*_s_(CH_2_) absorbance band of lipids and waxes is indicative of increased lipid or wax order as a result of tighter intermolecular packing and increased dispersion forces.^[^ ^29^, ^30^ ^]^ However, interpretation of FTIR reflectance spectra must be made with care. Variation in the reflectance signal is known to occur as function of surface topology and refractive index, which can result in shifts or distortions to absorbance bands.^[^ ^18^, ^20^, ^31^, ^32^ ^]^ Furthermore, it is unlikely that the spectra obtained are pure specular reflectance spectra, and some contribution from diffuse reflectance and transflection processes are probable,^[^ ^31^, ^32^, ^33^, ^34^ ^]^ which complicates in‐depth spectral interpretation. In addition, the reflectivity of the surface is governed by film thickness, angle of incidence of the light source, and molecular orientation.^[^ ^18^, ^19^, ^20^, ^21^ ^]^ Consequently, Lambert–Beer law is not obeyed, and direct measurement of relative concentration is difficult, if not impossible using the reflectance signal.^[^ ^21^ ^]^ Typically, in IRRAS the intensity and position is interpreted with respect to qualitative chemical information, such as molecular structure or orientation.^[^ ^18^, ^19^, ^20^, ^21^ ^]^ We anticipate that future work on this topic will enable more detailed information on the chemical composition and surface orientations of epicuticular waxes to be obtained, based on the peak positions and relative intensities observed in the reflectance spectra. At this stage however, regardless of the origin of the band shifts, their existence provide the ability to spatially and temporally resolve changes in the epicuticular wax layer, which in itself offers immense potential to study plant physiology.

The capability and benefits of the FTIR reflectance technique were further elucidated in a case study using wheat leaves infected with a devastating fungal pathogen, *Ptr*, which causes yellow spot disease. FTIR reflectance was able to reveal significant changes to the wheat epicuticular wax layer during the progression of disease. The wax layer directly on the surface of the necrotic tissue was impacted the most compared to the surrounding tissue and the control leaf samples. Interestingly, the regions adjacent to the necrotic tissue also showed significant spectroscopic changes despite no visible signs of leaf damage. This suggests that the epicuticular wax layer is dynamic to disease pressure. This also opens an avenue for detection of disease prior to appearance of visible symptoms. FTIR reflectance approach enables future mechanistic focused comprehensive studies in a broad range of interacting systems and the development of a new frontier for studying plant tolerance to disease.

## 4 Conclusion

We have demonstrated, for the first time, that FTIR reflectance microspectroscopic imaging technique promises the potential to be used as a non‐destructive chemical imaging tool for in situ and ex vivo or in vivo studies of plant epicuticular waxes. The FTIR reflectance spectra of the epicuticular wax layer vary with anatomical location on the plant leaf, and as a function of leaf maturation. Care must be taken when drawing scientific conclusions from FTIR reflectance spectra, to avoid misinterpretation of the chemical information that they contain. Future applications utilizing this method may include time‐course studies of the effects of various environmental stressors, such as disease, hyper‐salinity, or soil contamination to enable dynamic analysis of the epicuticular wax layer. Such studies have the potential to yield important physiological insights to optimize mitigation or rehabilitation strategies to counter environment stress, to optimize crop harvests, and inform breeding programs. In addition, the method may find use as a non‐destructive screening tool, to analyse large sample regions in order to identify smaller regions of interest for further analyses at higher spatial resolution using destructive techniques, such as ATR‐FTIR mapping or MS approaches.

## 5 Experimental Section

### The Preparation of *Eucalyptus* Leaf Samples—Eucalyptus gomphocephala

(Tuart) saplings were maintained in 9 L plastic potting containers filled with local soil (Swan coastal plain of Perth, Western Australia). Immature (<5 cm max length) and mature leaves (>10 cm max length) of 12‐month‐old saplings were harvested in early autumn (April 2018) or late winter (August 2018). The longitudinal study was performed in winter with samples harvested during August 1–21, 2018.

To investigate the effects of microtome sectioning, leaves were flash frozen in liquid nitrogen immediately after being removed from the *Eucalyptus* sapling, and stored at −80 °C until required for analysis. Leaves were sectioned to 5‐µm‐thick, using a Leica cryo‐microtome at −20 °C. The leaf sections were placed onto CaF_2_ windows and glass slides for transmission FTIR spectroscopic and Raman spectroscopic analyses, respectively.

### Preparation of Wheat Leaf Samples

Wheat (*Triticum aestivum* cv Scout) was utilized for the analysis of yellow spot disease caused by *Ptr*.^[^ ^22^ ^]^ Wheat seeds were sown in 2.1 L pots, and grown in Curtin University's glasshouse and maintained during the months of winter (June–August). The glasshouse temperatures ranged between 10 and 25 °C.

The pathogenic isolate of *Ptr*, race 1 isolate M4 (ToxA and ToxC producing) was utilized for the inoculation of wheat leaves. The isolate was cultured following the growth conditions previously described.^[^ ^35^ ^]^ Conidia were harvested in water and a 10 µL droplet containing ≈30 conidia was placed onto the adaxial side of leaf. Most recent mature leaf of 7 weeks old plants were used for inoculation limited to one leaf per plant. To help facilitate infection, the glasshouse humidity was maintained at ≥95% via a fitted misting system (Idrobase Fog Extra, Italy) for 48 h.

### Transmission Focal Plane Array ‐ Fourier Transform Infrared Spectroscopic Imaging of *Eucalyptus* Leaf Cryo‐Sections

FTIR microspectroscopic imaging of the leaf sections on CaF_2_ windows were analyzed at Infrared Microspectroscopy (IRM) beamline, Australian Synchrotron (Clayton, Australia), using an offline Bruker Hyperion 2000 FTIR microscope, equipped with a liquid‐N_2_ cooled 64 × 64 element FPA detector and a 15× objective lens (NA = 0.4), which was coupled to a Vertex 70 FTIR spectrometer (Bruker Optik GmbH, Ettlingen, Germany). Under this optical configuration, a single spectrum in each FTIR image represented molecular information acquired from ≈2.67 × 2.67 µm^2^ area on the sample plane. The spectral images were collected at 4 cm^−1^ spectral resolution, with 256 co‐added scans using OPUS 7.2 imaging software (Bruker). Background measurements were taken prior to sample spectral images, by focusing on a clean surface area of the CaF_2_ substrate using the same acquisition parameters.

### Raman Microspectroscopy of *Eucalyptus* Leaf Cryo‐Sections

Cryo‐sections of *Eucalyptus* leaf mounted onto glass microscope slides were used for Raman microspectroscopic analysis. The Raman maps were acquired using a WITec Alpha 300 SAR instrument, using 532 nm excitation laser, a 300 ms exposure time, with 250 nm step size and focus beam size of ≈300 nm at full width at half maximum (100× magnification objective).

### Synchrotron Attenuated Total Reflectance ‐ Fourier Transform Infrared Microspectroscopy of *Eucalyptus* Leaf

To facilitate ATR‐FTIR analysis of wax distribution on the surface of the *Eucalyptus* leaf, a 2 cm^2^ section of leaf was mounted on an aluminum disc with double sided sticky tape. The sample was then raised, so that the top (adaxial) side of the leaf was in contact with the ATR crystal.

The SR‐ATR‐FTIR measurements were performed at the Australian Synchrotron Infrared Microscopy (IRM) Beamline (Victoria, Australia), using the macro‐ATR accessory, which had previously been described.^[^ ^36^ ^]^ In brief, synchrotron macro ATR‐FTIR spectra were collected using a Bruker Vertex 80 v spectrometer coupled with a Hyperion 3000 FTIR microscope, equipped with a liquid nitrogen‐cooled narrow‐band mercury cadmium telluride detector (Bruker Optik GmbH, Ettlingen, Germany). The FTIR spectra were recorded within a spectral range of 3800–950 cm^−1^ using 8 cm^−1^ spectral resolution. Data collection was controlled using OPUS 7.2 software suite (Bruker Optik GmbH, Ettlingen, Germany). The macro ATR‐FTIR device was equipped with a germanium (Ge) ATR hemispherical crystal (*n*_Ge_ = 4.0), which had a 250 µm diameter active sensing area. The optics incorporate a 20× IR objective (NA = 0.60; Bruker Optik GmbH, Ettlingen, Germany). A background spectrum was recorded in air using 256 co‐added scans and the sample spectra were recorded using four co‐added scans once the contact between the ATR crystal and leaf sample was made. The sample was mapped with the beam focus size of 1.9 µm and an effective step size of 0.5 µm.

### Fourier Transform Infrared Reflectance Imaging Measurements

FTIR reflectance microspectroscopic imaging of *Eucalyptus* leaf tissue was carried out with a Nicolet iN 10MX FTIR microscope, an 8 × 2 pixel liquid nitrogen cooled linear array detector, and 25 µm pixel size. Spectra were collected using 4 cm^−1^ spectral resolution with 16 co‐added scans. A background reflectance image was collected from the surface of aluminum foil under the same conditions and using the same parameters. Strong FTIR reflectance spectra were confirmed to occur using either aluminum foil or a reflective gold surface for background reflection (Figure S2, Supporting Information). FTIR reflectance images of *Eucalyptus* leaf were also collected after ether extraction of waxes from the leaf surface (incubation of leaf in ditheyl ether for 20 min).

The optical microscopic images of leaves were collected prior to acquiring FTIR reflectance spectral images. The bright field capabilities of the FTIR microscope, were sufficient to identify regions of interest to image on the leaf. However, the quality of the bright field images was not sufficient to identify location of stomata. Bright field images of additional leaves from the same plant were collected at 4× and 10× magnification using an Olympus BX51 microscope with Olympus DP70 camera and cellSens Standard software.

FTIR reflectance images were collected from five replicates of immature and five mature Tuart leaves harvested in autumn (April 2018). FTIR reflectance images were also collected for an additional five mature leaves, harvested in late winter (August 2018). FTIR reflectance imaging parameters were as described above.

To demonstrate the capability of FTIR reflectance microspectroscopy for imaging the same leaf as a young and mature leaf, five leaves were imaged at two time points 7 weeks apart (April 8th, 2018 and May 28th, 2018). The average size of the young leaves was initially 3.0 cm in length and 0.7 cm in maximum width, which then extended to an average 8.6 cm long and 3.2 cm wide after 7 weeks. The leaf was taped flat to the motorized microscope mapping stage, using a small amount of adhesive tape gently placed over the tip and base of the leaf. FTIR reflectance imaging parameters were as described above.

### Scanning Electron Microscope Imaging of *Eucalyptus* Leaves

SEM images of *Eucalyptus* leaves were taken using benchtop SEM (Joel JCM‐6000 plus). An ≈1 cm^2^ section of leaf was deposited onto a SEM stub using carbon tape. The stub was coated with gold for 2 min using smart coater (DII‐29030SCTR) just before SEM imagining.

### Statistical Analysis

FTIR spectra were analyzed with Cytospec v2.00.03 and OPUS v7.0. The characteristic spectroscopic markers indicating the presence of wax were intense *ν*_as_(CH_2_) and *ν*_s_(CH_2_) absorbance modes with minimal or absent *ν*_as_(CH_3_), *ν*_s_(CH_3_), and olefinic *ν*(═C—H) modes.^[^ ^3^, ^37^ ^]^ False‐color functional group images of the epicuticular wax were generated from FTIR and Raman microspectroscopic data using the integrated area under the curve of the *ν*_s_(CH_2_) absorbance band (2865–2840 cm^−1^), or second derivative intensity at 2848 cm^−1^ (21‐smoothing point Savitzky–Golay derivatization function). FTIR reflectance spectra were not corrected using the Kramers–Kronig transformation, as the spectral shapes of the reflected signal indicated contributions from both specular, and diffuse reflectance.

Unless otherwise stated, FTIR spectra were presented as the raw data without preprocessing, or the data were presented as second‐derivatives. Second‐derivative spectra were derived using a 21‐smoothing point Savitzky–Golay derivatization function. Prior to data derivatization, spectra were vector normalized from 2800 to 3000 cm^−1^.

Statistical analysis was performed using the second‐derivative intensity or position of the *ν*_s_(CH_2_) absorbance band as the dependent variable, with independent variables of leaf maturity (immature or mature), season (autumn or winter), and anatomical location (stem, vein, tissue adjacent to stem, and tissue further from the stem). Each experimental group contained five replicates (*n* = 5) for all statistical testing. A two‐way repeated measures ANOVA was used to investigate the effect of seasonal change (Figure 2), leaf maturity (Figures 3 and 4) or disease state (Figure 5) with respect to the second‐derivative intensity or position of the *ν*_s_(CH_2_) absorbance band, as measured in specific anatomical locations on the leaf. If statistically significant effects were identified by the two‐way ANOVA, post‐hoc testing was undertaken. Post‐hoc testing was performed with *t*‐tests, corrected for four multiple comparisons using the Bonferroni method and an alpha of 0.05 (two‐tail testing). Statistical analysis was performed using GraphPad Prism v7.04. No outliers were removed in this study.

## Conflict of Interest

The authors declare no conflict of interest.

## Author Contributions

Conceptualization: G.S., F.N., A.P., M.R.G., M.J.H. Methodology: K.K., A.H., G.S., L.M.V.P.S., A.K., M.J.T., J.V., M.R.G., A.P., F.N., M.J.H. Investigation: K.K., A.H., G.S., L.M.V.P.S., A.K., M.J.T., J.V., M.R.G., A.P., F.N., M.J.H. Visualization: K.K., F.N., M.J.H. Supervision: F.N., A.P., M.R.G., M.J.H. Writing–original draft: K.K., F.N., J.V., M.J.H. Writing–review and editing: K.K., A.H., G.S., L.M.V.P.S., A.K., M.J.T., J.V., M.R.G., A.P., F.N., M.J.H.

## Supporting information

Supporting Information

## Data Availability

All data presented in the manuscript is available upon request from Curtin University's electronic data repository.
