# Supplementary material for: “Wax On, Wax Off”: In Vivo Imaging of Plant Physiology and Disease with Fourier Transform Infrared Reflectance Microspectroscopy
Source: Adv Sci (Weinh). 2021 Aug 2;8(19):2101902. doi: 10.1002/advs.202101902 (PMC8498906; doi:10.1002/advs.202101902)
Supplement: Supplementary file 1 — Supporting Information [file ADVS-8-2101902-s001.pdf]

© 2021 Wiley-VCH GmbH

ADVANCED SCIENCE
Open Access

Supporting Information

for *Adv. Sci.*, DOI: 10.1002/advs.202101902

In Vivo Imaging of Plant Physiology and Disease with FTIR Reflectance Microspectroscopy

*Karina Khambatta,[1] Ashley Hollings,[1] Georgina Sauzier,[1] Lilian M. V. P. Sanglard,[2] Annaleise Klein,[3] Mark J. Tobin,[3] Jitraporn Vongsvivut,[3] Mark R. Gibberd,[2] Alan Payne,[1] Fatima Naim,[2] Mark J. Hackett[1]**

Supplementary Materials for

"Wax On – Wax Off"

In Vivo Imaging of Plant Physiology and Disease with FTIR Reflectance Microspectroscopy

Karina Khambatta,[1] Ashley Hollings,[1] Georgina Sauzier,[1] Lilian M. V. P. Sanglard,[2] Annaleise Klein,[3] Mark J. Tobin,[3] Jitraporn Vongsvivut,[3] Mark R. Gibberd,[2] Alan Payne,[1] Fatima Naim,[2] Mark J. Hackett[1]*

*Corresponding author. Email: mark.j.hackett@curtin.edu.au

This PDF file includes:

Figs. S1 to S3

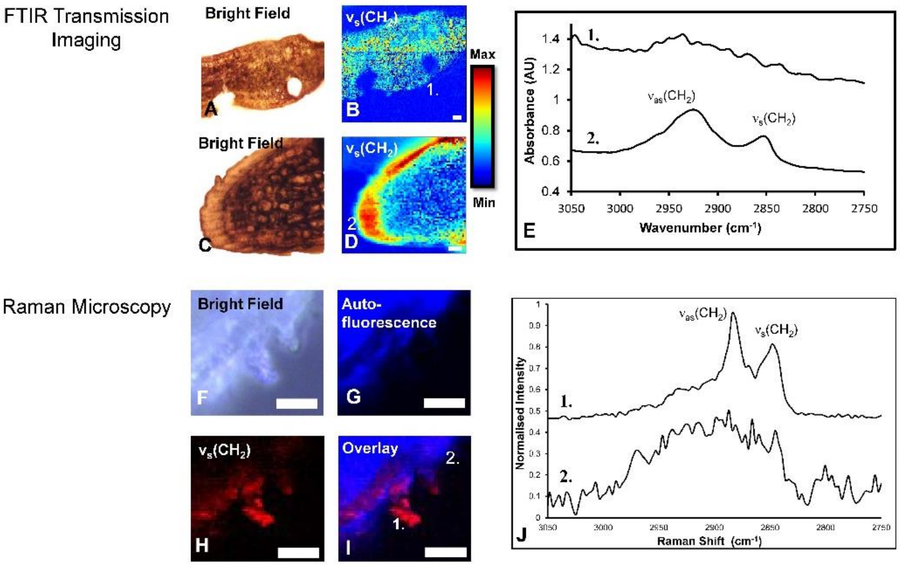

Figure. S1. The epicuticular wax layer is not consistently preserved in leaf sections, as observed through FTIR spectroscopic imaging (**A-E**), and Raman microscopy analysis (**F-J**). Bright field images reveal sample morphology (**A, C**). FTIR spectroscopic imaging revealed that the wax layer was absent in some sections (**B**), but present in other sections (**D**). False-colour functional group images in (**B)** and (**D)** were generated from integrated area under the curve for the $\nu_s(CH_2)$ absorbance band (2840 – 2865 cm^{-1}). Representative FTIR spectra from image positions 1 and 2 are shown in (**E)**. Raman microscopy analysis of leaf sections (**F-I**) yielded similar conclusions. The bright field image (**F**), and the autofluorscence Raman image reveals leaf structure (**G**), and the false-colour functional group images generated from integrated area under the curve for the $\nu_s(CH_2)$ absorbance band 2840 – 2865 cm^{-1} reveals location of epicuticular waxes (**H**). Overlay of the autofluorescence and epicuticular wax image reveals inconsistent preservation of epiculatular wax on the leaf surface (**I**). Representative Raman spectra from image positions 1 and 2 are shown in **J** .Scale bar F-I = 5 µm.

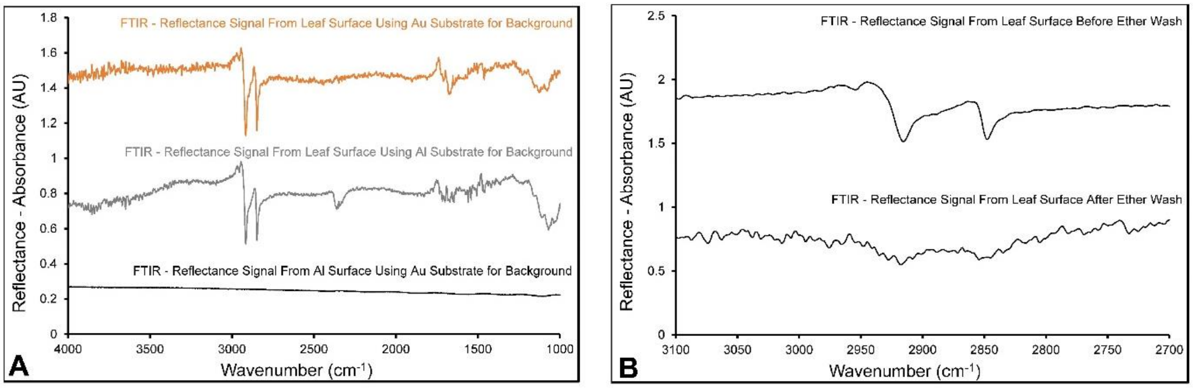

Figure. S2. (**A**) FTIR-reflectance signal of the epicuticular wax layer on the plant leaf surface is observed independent of background substrate used (Au vs Al). (**B**) Incubation of leaves in ether ("Ether Wash") extracts waxes from the leaf surface and drastically reduces the FTIR-reflectance signal.

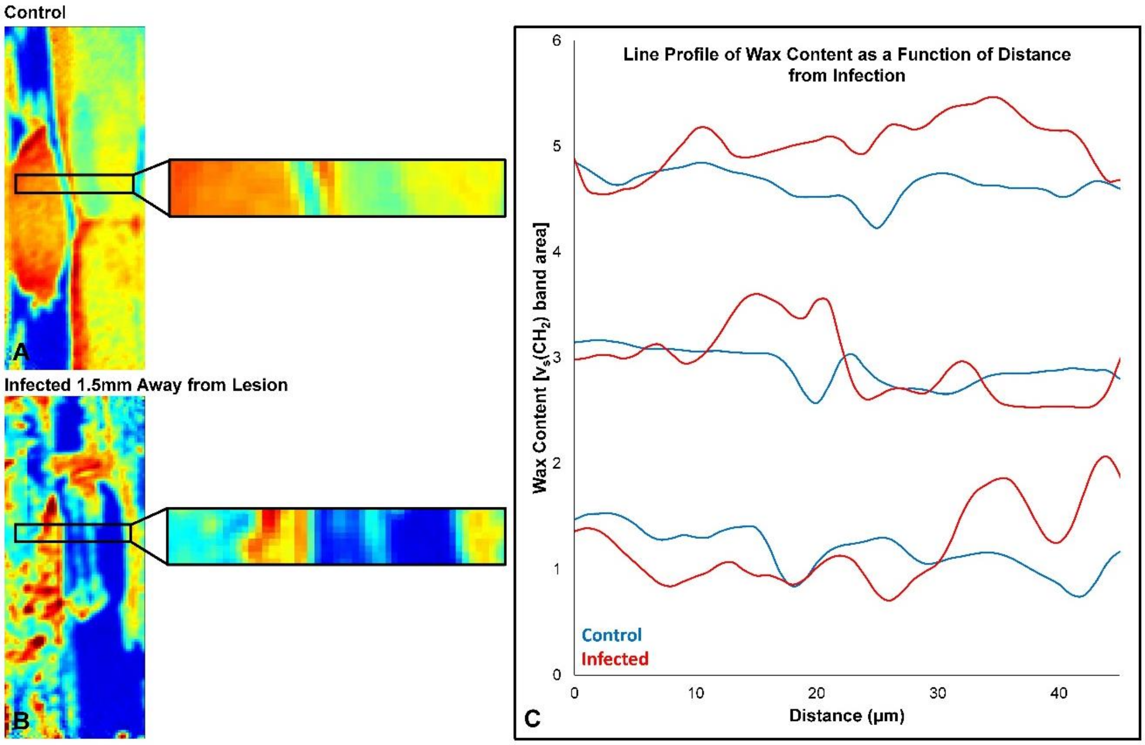

Figure. S3. Synchrotron-ATR reveals greater heterogeneity in the wax distribution across diseased wheat leaves, relative to healthy leaves. False-colour functional group images of the wax layer generated from areas under the curve of the $\nu_s(CH_2)$ absorbance bands 2830-2870 cm^{-1} for control (**A**) and infected leaf (scan taken 1.5 mm away from lesion) (**B**). Variability in wax content measured across a fixed distance can also be seen between control (blue) vs infected (red). Line plots for triplicate leaf measurements are shown (**C**). The images in A and B correspond to the middle set of line plots in C.
